# Oral remdesivir derivative VV116 is a potent inhibitor of respiratory syncytial virus with efficacy in mouse model

**DOI:** 10.1038/s41392-022-00963-7

**Published:** 2022-04-16

**Authors:** Ruxue Zhang, Yumin Zhang, Wei Zheng, Weijuan Shang, Yan Wu, Ning Li, Jun Xiong, Hualiang Jiang, Jingshan Shen, Gengfu Xiao, Yuanchao Xie, Leike Zhang

**Affiliations:** 1 State Key Laboratory of Virology, Wuhan Institute of Virology, Center for Biosafety Mega-Science, Chinese Academy of Sciences, Wuhan, 430071 China; 2 University of Chinese Academy of Sciences, Beijing, 100049 China; 3 Shanghai Institute of Materia Medica, Chinese Academy of Sciences, Shanghai, 201203 China; 4 Shanghai Junshi Biosciences Co., Ltd., Shanghai, 200126 China; 5 Lingang Laboratory, Shanghai, 200031 China

**Keywords:** Infection, Target validation

**Dear Editor,**

Respiratory syncytial virus (RSV) is the leading cause of serious lower respiratory tract disease in children under 5 years of age worldwide, causing an estimated 3.2 million hospitalizations and 120,000 deaths in children globally per year. Furthermore, nearly all children can be infected with RSV by 2 years of age, and individuals can be repeatedly re-infected with RSV throughout life, which poses great threats to infants, the elderly, and immunocompromised persons. At present, only palivizumab has been approved in the United States as a prophylactic treatment for the prevention of serious lower respiratory tract disease in children at high risk of RSV disease. However, palivizumab is only effective in ~50% of individuals in preventing hospitalization and the cost is prohibitive.^1^ Given the disease burden, RSV has been a priority for vaccine and antiviral drugs for over 50 years.

With over 30 drugs approved for the treatment of serious viral diseases, nucleotide and nucleoside analogs that function by targeting viral DNA or RNA polymerases represent one of the largest classes of antiviral drugs. β-d-N4-Hydroxycytidine and its prodrug EIDD-2801, which has been recently approved to treat COVID-19 in certain areas, also showed a strong inhibitory effect on RSV in vitro.^2^ Besides, ALS-8176 and its parent cytidine analog ALS-8112 have been discovered to be potent inhibitors of RSV replication but failed at Phase II clinical trial.^3^

Remdesivir (RDV), a phosphoramidate prodrug of 1′-CN-4-aza-7,9-dideazaadenosine C-nucleoside (GS-441524), has received great attention since the outbreak of SARS-CoV-2, and was formally approved by FDA for treating hospitalized patients with COVID-19. As a ProTide prodrug, RDV is administered intravenously because of extensive hepatic first-pass metabolism, which significantly limits its use. Notably, RDV is also identified as a potential treatment for RSV infection, but it still needs to be given by intravenous route.^4^ Recently, we reported an oral RDV derivative, VV116, which demonstrated potent anti-SARS-CoV-2 efficacy in hACE2-transduced Balb/c mice.^5^ VV116 is a tri-isobutyrate ester prodrug of the C7-deuterated GS-441524 analog (X1) (Fig. 1a), and is being investigated in Phase II/III clinical trials for the treatment of COVID-19. Herein, we reported that VV116 was a promising oral nucleoside antiviral against RSV infection.

**Fig. 1:**
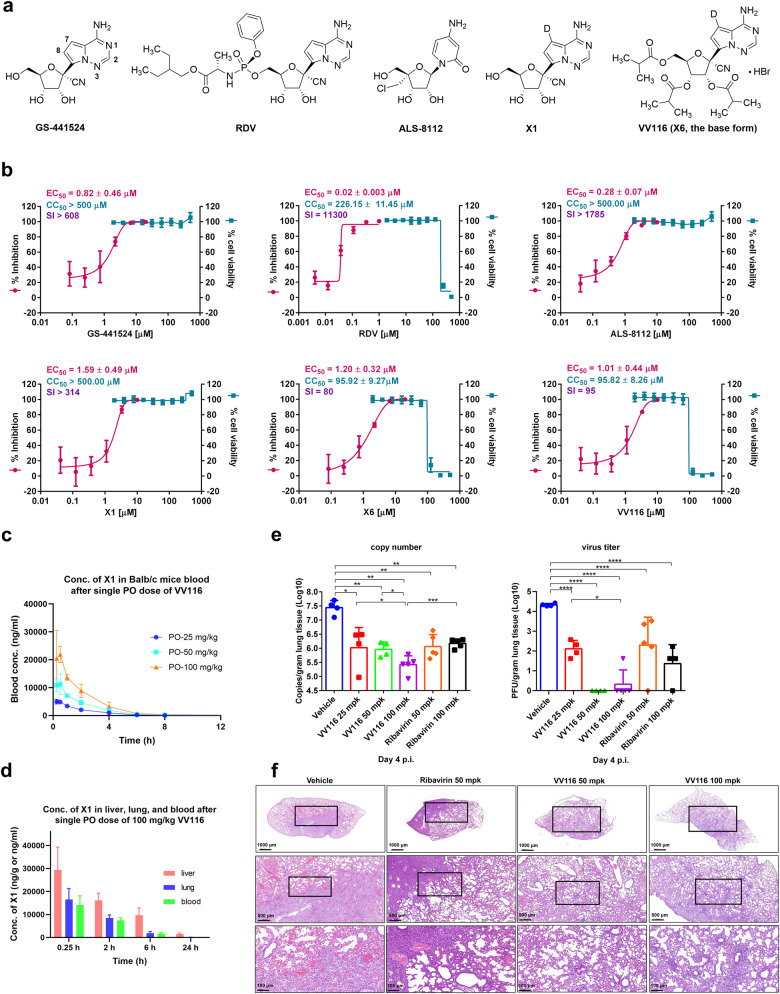
The oral RDV derivative, VV116, is an effective inhibitor of the respiratory syncytial virus. **a** The chemical structures of GS-441524, RDV, ALS-8112, the 7-deuterated GS-441524 analog X1, and the isobutyrate ester prodrug VV116. **b** Inhibition of RSV A2 replication and cellular toxicity of GS-441524, RDV, ALS-8112, X1, X6, and VV116 in A549 cells. *N* = 9 (three biological experiments with three technical replicates each). **c** Concentration of X1 in blood following oral administration of VV116 at a single dose of 25, 50, or 100 mg/kg in Balb/c mice (*N* = 3). **d** Concentration of X1 in liver, lung, and blood following oral administration of VV116 at a single dose of 100 mg/kg in Balb/c mice (*N* = 6). **e** Viral RNA and infectious titer levels in lung tissues of Vehicle-controlled, VV116- and Ribavirin- treated mice on day 4 post infection (p.i.). Error bars indicate SEM. mpk: mg/kg. The significance of the difference between mean values was determined by Student’s *t* test. *p < 0.05, **p < 0.005, ***p < 0.0005, ***p < 0.0001. **f** Histopathology of the lungs of the vehicle-controlled, VV116- and ribavirin-treated mice for 4 days. mpk: mg/kg

First, we determined the anti-RSV activities of GS-441524, RDV, X1, the base form of VV116 (X6), VV116, and ALS-8112 using A549 cells (Fig. 1a). GS-441524 and RDV had EC_50_s of 0.82 ± 0.46 μM and 0.02 ± 0.003 μM, respectively (Fig. 1b). X1 bearing a deuterium atom at the C7 position of the pyrrolotriazine base exhibited a similar antiviral activity (EC_50_ = 1.59 ± 0.49 μM, CC_50_ > 500 μM, SI > 314, EC_90_ = 3.56 ± 0.63 μM) to that of GS-441524. Previously, we found that X1 was not suitable for oral administration because of its low oral bioavailability in rats (*F* = 21.7%). Utilizing the ester prodrug approach that could improve intestinal permeability,^4^ we developed a tri-isobutyrate ester prodrug, X6, and this ester was subjected to salt formation to afford the hydrobromide salt (VV116) that was identified as the optimal drug candidate. VV116 had high oral bioavailability in preclinical species with F values of ~80% in rats and ~90% in dogs,^5^ and in ICR mice with F value of 110.2% (Fig. 1c, d, Supplementary Table S1, S2). Moreover, we discovered that VV116 had pharmacokinetic (PK) advantages relative to its non-deuterated counterpart in SD rats (supplementary Tables S3–S5). The tri-isobutyrate ester VV116 can also inhibit RSV replication (EC_50_ = 1.20 ± 0.32 μM, CC_50_ = 95.92 ± 9.27 μM, SI = 80, EC_90_ = 3.08 ± 1.253 μM) in A549 cells, which suggested that the ester moiety of VV116 was susceptible to hydrolysis by cellular enzymes to release the parent nucleoside. Anti-RSV activities of these compounds were also confirmed in HEp-2 and NHBE cells, other permissive cells for RSV (supplementary Fig S1, S2).

Considering the potent effect of VV116 inhibiting RSV in vitro, we further tested the effect of VV16 against RSV in a mouse model. Ribavirin, the off-label used drug to treat RSV in the clinic, was employed as a control. To this end, 6–8-week Balb/c mice were intranasally infected with 4 × 10^6^ FFU of RSV per mouse (day 0), and were then treated with VV116 (25, 50, and 100 mg/kg) or ribavirin (50 and 100 mg/kg) bis in die (b.i.d.) (supplementary Fig S3). Our previous study indicated that both viral load and pathology reached high in RSV infected mice at day 4 post infection (p.i.), and hence at this time point, mice were killed, and lungs were fetched. Viral RNA level in the lung was measured with quantitative RT-PCR and virion load was measured with immunoplaque assay (Fig. 1e). Of note, the low dose of VV116 (25 mg/kg) displayed a comparable antiviral effect to that of 100 mg/kg of ribavirin, which decreased the viral RNA copies and the infectious tilters by ~1.5 log10 and ~2.0 log10, respectively (Fig. 1e). The medium dose (50 mg/kg) of VV116 exhibited a stronger activity and decreased the virus titers below the detection limit (Fig. 1e). We also evaluated the lung pathology of the challenged mice by histochemical analysis. After RSV infection, mice treated with vehicle displayed severe inflammation with alveolar inflammatory patches. By contrast, only slight lung infiltration was observed in mice treated with VV116, demonstrating that VV116 treatment can reduce lung injury after RSV infection (Fig. 1f, Supplementary Fig S4).

The PK study in Balb/c mice showed that VV116 had a linear PK profile in doses of 25 to 100 mg/kg (Fig. 1c, supplementary Table S6). Because of the first-pass metabolism of the esterase-sensitive prodrug, VV116 was not detected in mouse plasma even at 100 mg/kg. Following oral administration, the blood concentration of the parent nucleoside X1 quickly reached C_max_ within 0.5 h, and at the dose of 25 mg/kg, the mean C_max_ reached 5360 ng/ml (18.4 µM, Fig. 1c, supplementary Table S6, S7), which was much higher than the EC_90_ value in vitro. X1 had a short elimination half-life (2.3–4.25 h, supplementary Table S6), which supported a twice-daily dosing regimen. The ester prodrug form of VV116 was designed not only for improving oral adsorption but to circumvent the liver-targeting issue of the nucleoside phosphoramidate prodrugs. The preclinical tissue distribution study revealed that X1 was widely distributed in SD rat tissues,^5^ and a favorable distribution of X1 was also observed in Balb/c mice with the concentration of X1 in the lung being about half of that in the liver (Fig. 1d, supplementary Table S8). With respect to the therapeutic window of VV116, the 14-day repeated dose oral toxicity study in rats revealed a NOAEL (No-Observed-Adverse-Effect-Level) of 200 mg/kg, at which the AUC_0–t_ of X1 reached a value of 85151 ng h/ml (Supplementary Table S9), ~3.5-folds of that at the dose of 50 mg/kg in mice.

Nucleoside antiviral agents have a high genetic barrier to resistance since they target the highly conserved catalytic center of viral polymerase, and VV116 has been found to be effective against different SARS-CoV-2 variants. The favorable PK properties and good safety profile make it to be a very promising oral antiviral for treating COVID-19. Herein, the in vivo efficacy study also provided strong evidence for potential therapeutic usage of VV116 against RSV infection. The clinical studies of VV116 should be considered to mitigate RSV infection.

## Supplementary information

Supplementary information

## Data Availability

Materials are available upon request.
