# Supplementary material for: Oral remdesivir derivative VV116 is a potent inhibitor of respiratory syncytial virus with efficacy in mouse model
Source: Signal Transduct Target Ther. 2022 Apr 16;7:123. doi: 10.1038/s41392-022-00963-7 (PMC9012943; doi:10.1038/s41392-022-00963-7)
Supplement: Supplementary file 1 — Supplementary information [file 41392_2022_963_MOESM1_ESM.docx]

Supplementary Materials for

**Oral remdesivir derivative VV116 is a potent inhibitor of respiratory syncytial virus with efficacy in a mouse model**

Ruxue Zhang^1,3*^, Yumin Zhang^1*^, Wei Zheng^2,3*^, Yan Wu^1^, Jun Xiong^4^, Hualiang Jiang^2,3^, Jingshan Shen^2,3^, Gengfu Xiao^1,3^, Yuanchao Xie^2,5#^, Leike Zhang^1,3#^

^1^ State Key Laboratory of Virology, Wuhan Institute of Virology, Center for Biosafety Mega-Science, Chinese Academy of Sciences, Wuhan, 430071, China

^2^ Shanghai Institute of Materia Medica, Chinese Academy of Sciences, Shanghai, 201203, China

^3^ University of Chinese Academy of Sciences, Beijing, 100049, China

^4^ Shanghai Junshi Biosciences Co., Ltd. Shanghai, 200126, China

^5^ Lingang Laboratory, Shanghai, 200031, China

**This PDF file includes:**

Materials and Methods

Figures S1 to S4

Tables S1 to S9

Materials and Methods

**Cells and viruses**

All the cells used in this study were cultured in humidified incubator under 37℃ with 5% CO_2_. Human laryngeal epidermoid carcinoma (HEp-2) cells, Vero E6 cells, and A549 cells were grown in Dulbocco’s Medified Eagle Medium (DMEM; Gibco), supplemented with 10% fetal bovine serum (FBS; Glibco). Normal human bronchial epithelial (NHBE) cells were maintained in Bronchial Epithelial Cell Growth Medium (BEGM) with all provided supplements in the BulletKit.

RSV A2 strain was grown in HEp-2 cells. At 3 or 4 days post-infection, viruses were collected from infected cells. Briefly, RSV infected cells were repeated freezing and thawing 3 times, then the cells were centrifuged at 1000 rpm for 10 min at 4℃. Afterwards, the supernatant was collected and stored at -80℃ until used. Viral titer of RSV A2 was determined in Vero E6 cells by immunoplaque assay as described previously^[1](#_ENREF_1)^. All the RSV A2 infection experiments were carried out in biosafety level-2 (BSL-2) laboratory.

**Antiviral activities and cytotoxicity measurement**

A549, HEp-2 or NHBE cells were plated into 48 well-plate and incubated overnight. Upon reaching 80% cell confluence, cells were infected RSV A2 (at an MOI of 2 in A549, MOI of 0.5 in HEp-2, and MOI of 1 in NHBE cells) for 2 h after cells were incubated for 1 h with varying concentrations of drugs. Then, the virus-drug mixture was removed, and cells were cultured with drug-containing medium. At 48 h post inoculation, total RNAs were extracted from cells and then in reverse transcription using PrimeScript RT reagent Kit with gDNA Eraser (TaKaRa). For determining the viral copies, absolute quantitative RT-PCR was performed with TB Green® Premix Ex Taq^TM^ II (TaKaRa). RSV A2 F fragment was quantified with primers 5’-CGAGCCAGAAGAGAACTACCA-3’; and 5’-CCTTCTAGGTGCAGGACCTTA-3’

Cell viability was performed in 96-well plate with triplicate for each concentration. All drugs were diluted 2 times with 9 gradients starting at 500 micromores in maintenance medium (DMEM containing 2% FBS). After 48 h incubation, the supernatant was removed, and 10 μL WST-8 (2-(2-methoxy-4-(phenyl)-3-(4-(phenyl) to 5 (2, 4-sulpho benzene) -2 h-tetrazolium monosodium salt) in maintenance medium was added in medium. Plates were measured at 450 nm wavelength using spectrophotometer (BioTek) after 2 h incubation, and cell viability was calculated.

***In vivo* efficacy of VV116 against RSV in mice**

Specific pathogen-free (SPF) female Balb/c mice at the age of 6–8 weeks were purchased from Beijing Vital River Laboratory Animal Technology Co., Ltd. The mice were housed in an SPF environment under standard conditions. All mouse experiments were approved by the ethics committee of the Wuhan Institute of Virology, Chinese Academy of Science (permit number WIVA25202113). Thirty mice (5 animals per group, 6 groups) were anaesthetized with isoflurane and challenged with 4×10^6^ FFU of RSV A2 intranasally (i.n.). The mice were given drugs by intragastric administration. Treatments were commenced in 1 h post infection and continued for 4 days. Mice in the control group were given the solvent (40% PEG 400+10% HS 15+50% ultrapure water (v:v:v)). The mice were euthanized on the 4th day after challenge and their lungs were collected. The weight of the mice was recorded daily.

The left lung was fixed in tissue fixative solution, embedded, sectionized and stained with H&E to observe the pathological changes of lung tissue. After weighing the right lung, add 400μL PBS into the tube, grind it with a grinding instrument. One part of the grinding tissue was used to determine the virus titer, and the other part was used to determine virus copy number by extracted RNA from the tissue supernatant using viral DNA/RNA extraction Kit (TaKaRa, 9766). The determination of viral titers and subsequent treatment of the RNA obtained were the same as above.

**Pharmacokinetic study of VV116 in ICR mice, Balb/c mice, and SD rats**

All the PK studies were conducted at Suzhou HQ Bioscience Co., Ltd.

ICR mice (N = 3 for each group, male) were fasted for 12 h before dosing (only for the oral administration). VV116 dissolved in DMSO-enthanol-PEG300-saline (5/5/40/50, v/v/v/v) was administered intravenously at 5.0 mg/kg, and orally at 25 mg/kg. At 5 min, 0.25, 0.5, 1.0, 2.0, 4.0, 6.0, 8.0, and 24 h post-dosing, blood samples were collected from the jugular vein or the submandibular vein into EDTA-K2 tubes, and immediately mixed with acetonitrile (20 µL blood + 80 μL acetonitrile). The concentrations of analytes in the blood were analyzed by LC-MS/MS.

A total of nine Balb/c mice (N = 3 for each group, male) were divided into three groups, and fasted for 12 h before dosing. The three groups received oral dose of VV116 dissolved in 40%PEG400+10% Kolliphor® HS15+50% ultrapure water at 25 mg/kg, 50 mg/kg and 100 mg/kg, respectively. At 5 min, 0.25, 0.5, 1.0, 2.0, 4.0, 6.0, 8.0, and 24 h post-dosing, blood samples were collected from the jugular vein or the submandibular vein into EDTA-K2 tubes, and immediately mixed with acetonitrile (20 µL blood + 80 μL acetonitrile). The concentrations of analytes in the blood were analyzed by LC-MS/MS.

SD rats (N = 3 for each group, male) were fasted for 12 h before dosing (only for the oral administration). The test compound (VV116 or VV116-H) was administered intravenously at 5.0 mg/kg dissolved in DMSO-enthanol-PEG300-saline (5/5/40/50, v/v/v/v), and administered orally at 30 mg/kg dissolved in 40%PEG400+10% Kolliphor® HS15+50% ultrapure water. At 5 min, 0.25, 0.5, 1.0, 2.0, 4.0, 6.0, 8.0, and 24 h post-dosing, blood samples were collected from the jugular vein into EDTA-K2 tubes. Serum samples were obtained following general procedures and the concentrations of analytes in the supernatant were analyzed by LC-MS/MS.

**Tissue distribution study of VV116 in Balb/c mice**

A total of thirty Balb/c mice were divided into five groups (3 animals/sex/group). VV116 was intragastrically administered at 100 mg/kg dissolved in 40%PEG400+10% Kolliphor® HS15+50%. At 0 (not administered), 0.25, 2, 6, and 24 h post-dosing, the five groups of mice were anesthetized, respectively. Blood samples were collected, and tissues including liver and lung were harvested. Tissue samples were individually homogenized, and blood samples were processed as above. The concentrations of X1 in liver, lung and blood were analyzed by LC-MS/MS.

**Genetic toxicity assay**

The Ames test, the rat micronucleus assay, and the chromosome aberration test were conducted according to NMPA and ICH guidelines.

The Ames test was conducted to determine the mutagenicity of VV116 using histidine-dependent Salmonella typhimurium (TA97a, TA98, TA100, TA1535) and tryptophan-dependent Escherichia coli (WP2). The experiment was carried out by plate permeating method under the -S9 non-metabolic and +S9 metabolic activation conditions. There were 6 dose groups for VV116 (5, 50, 150, 500, 1500 and 5000 µg/dish under each condition) with the negative control (DMSO) and positive controls (ICR191, 2-nitrofluorene, sodium azide, 2-aminofluorene and methyl methanesulfonate). Under the conditions of -S9 and +S9, the average numbers of revertant colonies in the positive control group of each strain were at least twice that of the negative control group. The numbers of revertant colonies of each strain in all VV116 dose groups were less than twice that of the negative control group, and did not show dose-dependent increase. The result showed that VV116 was not mutagenic to histidine-dependent Salmonella typhimurium and tryptophan-dependent Escherichia coli.

The chromosome aberration test was conducted to evaluate whether VV116 had the effect of inducing chromosome damage in Chinese hamster lung (CHL) cells by determining the aberration rate (excluding chromosome gap) under the -S9 and +S9 conditions. CHL cells were exposed to VV116 without S9 for 4 h at the concentrations of 10, 20, 35, 40, 43, 45 and 48 μg/mL (-S9/4h group), or 24 h at the concentrations of 5, 10, 20, 25, 30, 35 and 40 μg/mL (-S9/24h group). In the presence of S9 mix, CHL cells were treated with VV116 for 4 h at the concentrations of 10, 25, 50,100 and 150 μg/mL (+S9/4h group). Meanwhile, negative (DMSO), and positive control groups (Mitomycin C and cyclophosphamide monohydrate) were set up. Based on the cytotoxicity of VV116, three doses of each group were chosen for chromosome aberration analysis. The positive compounds obviously induced chromosome aberrations compared with the negative control. For the -S9/4h group of VV116, the chromosome aberration rates at the concentrations of 20, 35 and 40 µg/mL were 0.0%, 0.3% and 0.0%, respectively; For the -S9/24h group, the rates at the concentrations of 10, 25 and 30 µg/mL were 1.0%, 0.3% and 0.3%, respectively. And for the +S9/4h group, the rates at the concentrations of 20, 50 and 150 µg/mL were 1.3%, 0.7% and 0.3%, respectively. The chromosome aberration rates of all VV116 groups were within the background range, and showed no statistical difference compared with that of the negative control group. The result indicated that VV116 had no effect of inducing chromosome aberration in CHL cells.

The micronucleus assay in rats was conducted to evaluate whether VV116 has the effect of inducing any increase of micronucleated polychromatic erythrocytes in rat bone marrow. Groups of male and female SD rats (5 animals/sex/group) received oral doses of VV116 at 0 (vehicle control), 100 (low), 200 (mild) and 500 mg/kg/d (high) for 14 days. The animals were sacrificed within 24 h after the last dose. Bone marrow smears were prepared for examining the ratio of polychromatic erythrocyte/(polychromatic erythrocyte + normochromatic erythrocyte) (PCE/(PCE + NCE)) and the micronucleus rate of polychromatic erythrocytes (MnPCE/PCE). The result showed that the PCE/(PCE + NCE) ratios of the female animals of the vehicle group, the low, the mild, and the high dose VV116 group were 0.65, 0.57, 0.58 and 0.58, respectively. For the male animals, the ratios were 0.62, 0.64, 0.66 and 0.60, respectively. VV116 did not show obvious bone marrow toxicity in rats. The assay was valid as the average micronucleus rates were 1.4‰ and 0.7‰ for the female and male rats in the vehicle group, respectively, which were within the historical range. The micronucleus rates of the female animals of the three VV116 groups were 1.2‰, 1.0‰ and 0.7‰, respectively, and for the male animals, the rates were 0.3‰, 0.7‰ and 0.3‰, respectively. There was no effect of any dose of VV116 on the micronucleus rate compared to the negative control. VV116 did not have the effect of inducing the increase of micronucleated polychromatic erythrocytes in rat bone marrow up to 500 mg/kg/d for 14 days.

**Toxicokinetics of VV116 in SD rats**

Groups of male and female SD rats (4 animals/sex/group) received repeated oral doses of VV116 (dissolved in 40%PEG400+10% Kolliphor® HS15+50% ultrapure water) at 100 (low), 200 (mild) and 500 mg/kg/d (high) for 14 days. At day 1 and day 14, blood samples were collected from the jugular vein into EDTA-K2 tubes at various time points post-dose. Plasma samples were obtained following general procedures and the concentrations of analytes in the samples were analyzed by LC-MS/MS.

**Statistics**

All cell and animal data analyses and graphs were performed using GraphPad Prism software version 8 (GraphPad Software Inc., San Diego, CA).

**References**

1 Zhang Y. *et al.* A novel RSV F-Fc fusion protein vaccine reduces lung injury induced by respiratory syncytial virus infection. *Antiviral research.***165**:11-22, **(**2019**)**

**Supplementary Figures**

**Figure S1. Inhibition of RSV A2 replication and cellular toxicity of GS-441524, RDV, ALS-8112, X1, X6, and VV116 in HEp-2 cells.** N=6 (two biological experiments with three technical replicates each).

**

**

**Figure S2. Inhibition of RSV A2 replication and cellular toxicity of GS-441524, RDV, ALS-8112, X1, X6, and VV116 in human bronchial epithelial (NHBE) cells.** N=6 (two biological experiments with three technical replicates each).

**

**

**Figure S3. The design of the *in vivo* anti-RSV efficacy test of VV116 in mouse model.**

**
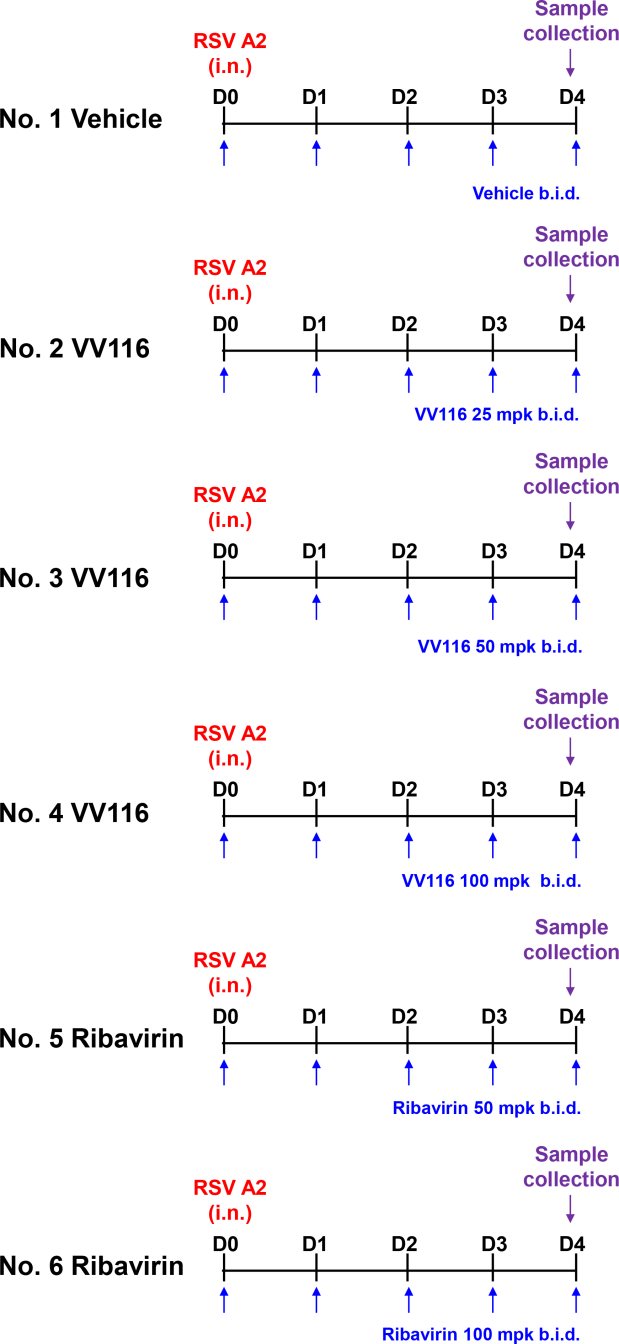
**

**Figure S4. Histopathology of the lungs of the Vehicle-controlled, VV116 25 mpk- and Ribavirin 100 mpk- treated mice for 4 days. mpk: mg/kg.**

**
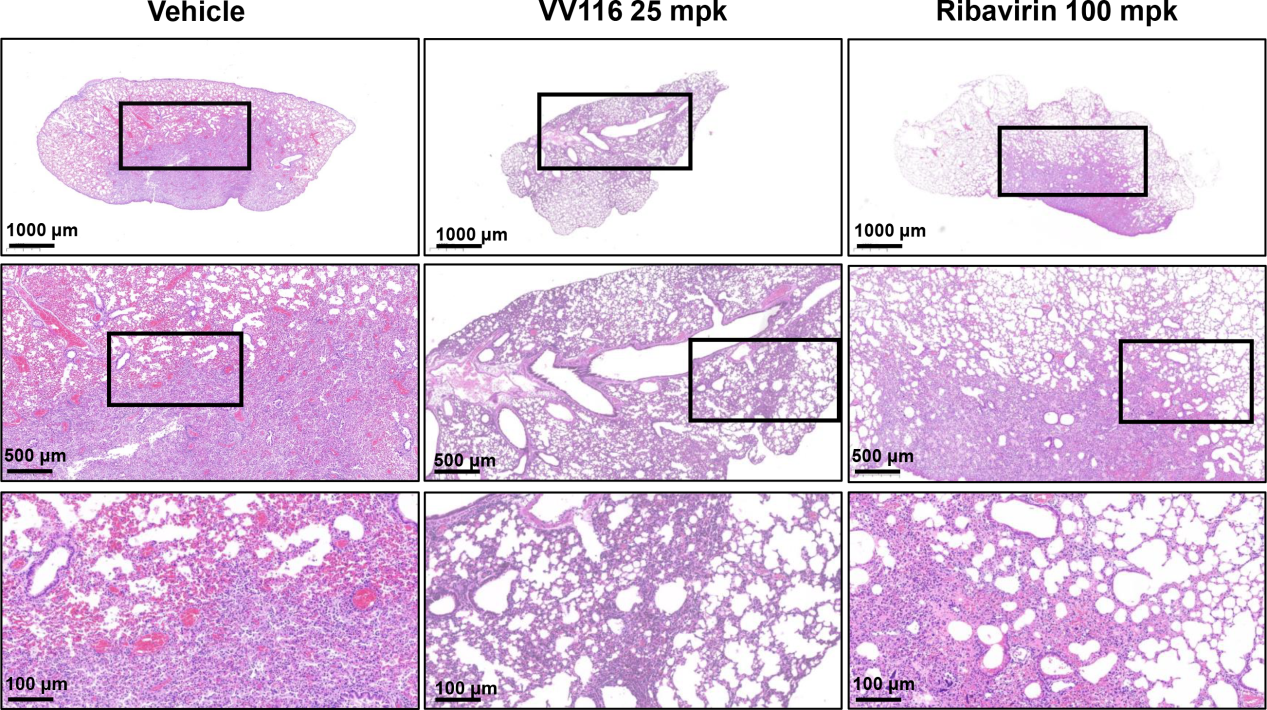
**

**Supplementary Table**

**Table S1**. **Single-dose PK parameters for X1 in mice.** Calculation of PK parameters for X1 following oral administration of VV116 at a dose of 25.0 mg/kg, and intravenous administration at a dose of 5.0 mg/kg in ICR mice (N = 3 per group).

| **Compd.**  **(Route)** | **T_max_** | **C_max_** | **AUC_0-t_** | **AUC_0-∞_** | **MRT_0-∞_** | **T_1/2_** | **CL** | **Vss** | **F** |
| --- | --- | --- | --- | --- | --- | --- | --- | --- | --- |
|  | **(h)** | **(ng/mL)** | **(h*ng/mL)** | **(h*ng/mL)** | **(h)** | **(h)** | **(mL/min/kg)** | **(L/kg)** | **(%)** |
| VV116  (p.o.) | 0.33 ± 0.14 | 6500 ± 529 | 12774 ± 1535 | 12868 ± 1588 | 2.90 ± 0.34 | 4.50 ± 0.89 | - | - | 110.2 |
| VV116  (i.v.) | - | 2995 ± 188 | 2312 ± 149 | 2341 ± 144 | 0.93 ± 0.17 | 1.57 ± 0.98 | 35.7 ± 2.2 | 1.99 ± 0.38 |  |

**Table S2**. **Concentration of X1 in blood of mice.** Concentration of X1 in blood following oral administration of VV116 at a dose of 25.0 mg/kg, and intravenous administration at a dose of 5.0 mg/kg in ICR mice (N = 3 per group).

| VV116, IV, 5.0 mg/kg | | | | | | VV116, PO, 25.0 mg/kg | | | | | |
| --- | --- | --- | --- | --- | --- | --- | --- | --- | --- | --- | --- |
| Time | Mice 1 | Mice 2 | Mice 3 | Mean | SD | Time | Mice 4 | Mice 5 | Mice 6 | Mean | SD |
|  | Con. (ng/ml) | | | | |  | Con. (ng/ml) | | | | |
| 5 min | 3175 | 3010 | 2800 | 2995 | 188 | 0.25 h | 6100 | 6300 | 5000 | 5800 | 700 |
| 0.25 h | 1870 | 2140 | 2110 | 2040 | 148 | 0.5 h | 4085 | 4210 | 7100 | 5132 | 1706 |
| 0.5 h | 1445 | 1335 | 1360 | 1380 | 58 | 1.0 h | 2875 | 2495 | 2645 | 2672 | 191 |
| 1.0 h | 685 | 580 | 640 | 635 | 53 | 2.0 h | 2745 | 1605 | 1875 | 2075 | 596 |
| 2.0 h | 236 | 136 | 150 | 174 | 54 | 4.0 h | 1560 | 1275 | 1485 | 1440 | 148 |
| 4.0 h | 50.5 | 37.7 | 28.1 | 38.8 | 11.2 | 6.0 h | 274 | 186 | 216 | 225 | 45 |
| 6.0 h | 13.5 | 25.1 | 10.6 | 16.4 | 7.7 | 8.0 h | 100 | 77.5 | 74.0 | 83.8 | 14.1 |
| 8.0 h | BQL | 13.5 | BQL | 13.5 | / | 24.0 h | 38.2 | 11.6 | 13.5 | 21.1 | 14.9 |
| 24.0 h | BQL | BQL | BQL | / | / |  |  |  |  |  |  |

BQL = below the quantification limit

**Table S3**. **Single-dose PK parameters for X1 or GS-441524 in rats.** Calculation of PK parameters for X1 or GS-441524 following oral administration of VV116 or VV116-H at a dose of 30.0 mg/kg, and intravenous administration at a dose of 5.0 mg/kg in SD rats (N = 3 per group).

| **Compd.**  **(Route)** | **T_max_** | **C_max_** | **AUC_0-t_** | **AUC_0-∞_** | **MRT_0-∞_** | **T_1/2_** | **CL** | **Vss** | **F** |
| --- | --- | --- | --- | --- | --- | --- | --- | --- | --- |
|  | **(h)** | **(ng/mL)** | **(h*ng/mL)** | **(h*ng/mL)** | **(h)** | **(h)** | **(mL/min/kg)** | **(L/kg)** | **(%)** |
| VV116  (p.o.) | 0.25 ± 0.0 | 2710 ± 738 | 9259 ± 1652 | 9407 ± 1581 | 3.82 ± 0.48 | 5.18 ± 1.71 | - | - | 87.0 |
| VV116  (i.v.) | - | 1923 ± 215 | 1774 ± 179 | 1802 ± 197 | 0.83 ± 0.07 | 0.67 ± 0.10 | 46.6 ± 4.8 | 2.31 ± 0.06 |  |
| VV116-H  (p.o.) | 1.00 ± 0.87 | 2060 ± 520 | 7664 ± 802 | 7768 ± 827 | 3.47 ± 0.80 | 4.59 ± 2.08 | - |  | 75.6 |
| VV116-H  (i.v.) | - | 1997 ± 81 | 1689 ± 80 | 1709 ± 74 | 0.82 ± 0.05 | 0.73 ± 0.07 | 48.8 ± 2.1 | 2.41 ± 0.05 |  |

**Table S4**. **Concentration of X1 or GS-441524 in rat plasma.** Concentration of X1 or GS-441524 in plasma following intravenous administration of VV116 or VV116-H at a single dose of 5.0 mg/kg in SD rats (N = 3 per group).

| VV116, IV, 5.0 mg/kg | | | | | | VV116-H, IV 5.0 mg/kg | | | | | |
| --- | --- | --- | --- | --- | --- | --- | --- | --- | --- | --- | --- |
| Time | Rat 1 | Rat 2 | Rat 3 | Mean | SD | Time | Rat 4 | Rat 5 | Rat 6 | Mean | SD |
|  | Con. (ng/ml) | | | | |  | Con. (ng/ml) | | | | |
| 5 min | 1820 | 2170 | 1780 | 1923 | 215 | 5 min | 1940 | 1960 | 2090 | 1997 | 81 |
| 0.25 h | 1440 | 1730 | 1590 | 1587 | 145 | 0.25 h | 1560 | 1620 | 1490 | 1557 | 65 |
| 0.5 h | 1030 | 1110 | 1040 | 1060 | 44 | 0.5 h | 1040 | 921 | 988 | 983 | 60 |
| 1.0 h | 505 | 609 | 579 | 564 | 54 | 1.0 h | 425 | 522 | 558 | 502 | 69 |
| 2.0 h | 162 | 219 | 153 | 178 | 36 | 2.0 h | 150 | 163 | 162 | 158 | 7 |
| 4.0 h | 18 | 42.6 | 20.9 | 27 | 13 | 4.0 h | 23.6 | 24.3 | 27.8 | 25 | 2 |
| 6.0 h | BQL | BQL | BQL | / | / | 6.0 h | BQL | BQL | 11.2 | 11 | / |
| 8.0 h | BQL | BQL | BQL | / | / | 8.0 h | BQL | BQL | BQL | / | / |
| 24.0 h | BQL | BQL | BQL | / | / | 24.0 h | BQL | BQL | BQL | / | / |

BQL = below the quantification limit

**Table S5**. **Concentration of X1 or GS-441524 in rat plasma.** Concentration of X1 or GS-441524 in plasma following oral administration of VV116 or VV116-H at a single dose of 30.0 mg/kg in SD rats (N = 3 per group).

| VV116, PO, 30.0 mg/kg | | | | | | VV116-H, PO, 30.0 mg/kg | | | | | |
| --- | --- | --- | --- | --- | --- | --- | --- | --- | --- | --- | --- |
| Time | Rat 1 | Rat 2 | Rat 3 | Mean | SD | Time | Rat 4 | Rat 5 | Rat 6 | Mean | SD |
|  | Con. (ng/ml) | | | | |  | Con. (ng/ml) | | | | |
| 5 min | 625 | 787 | 569 | 660 | 113 | 5 min | 122 | 279 | 353 | 251 | 118 |
| 0.25 h | 3190 | 3080 | 1860 | 2710 | 738 | 0.25 h | 1450 | 1180 | 1810 | 1480 | 316 |
| 0.5 h | 2420 | 2400 | 1710 | 2177 | 404 | 0.5 h | 1790 | 1440 | 2660 | 1963 | 628 |
| 1.0 h | 1660 | 2400 | 1620 | 1893 | 439 | 1.0 h | 1570 | 1490 | 1510 | 1523 | 42 |
| 2.0 h | 2110 | 1810 | 1560 | 1827 | 275 | 2.0 h | 1610 | 1730 | 1810 | 1717 | 101 |
| 4.0 h | 1040 | 762 | 775 | 859 | 157 | 4.0 h | 761 | 995 | 825 | 860 | 121 |
| 6.0 h | 285 | 496 | 176 | 319 | 163 | 6.0 h | 154 | 165 | 218 | 179 | 34 |
| 8.0 h | 114 | 189 | 72.8 | 125 | 59 | 8.0 h | 32.8 | 90 | 106 | 76 | 38 |
| 24.0 h | 12.3 | 21.7 | 23.1 | 19 | 6 | 24.0 h | 9.79 | 23.3 | 6.27 | 13 | 9 |

**Table S6**. **Single-dose PK parameters for X1 in Balb/c mice.** Calculation of PK parameters for X1 following oral administration of VV116 at a dose of 25.0, 50.0 and 100.0 mg/kg in Balb/c mice (N = 3 per group).

| **Compd.** | **Dose** | **T_max_** | **C_max_** | **AUC_0-t_** | **AUC_0-∞_** | **MRT_0-∞_** | **T_1/2_** |
| --- | --- | --- | --- | --- | --- | --- | --- |
|  | **(mg/kg)** | **(h)** | **(ng/mL)** | **(h*ng/mL)** | **(h*ng/mL)** | **(h)** | **(h)** |
| VV116  (p.o.) | 25 | 0.42 ± 0.14 | 5360 ± 560 | 11461 ± 1013 | 11534 ± 992 | 2.25 ± 0.32 | 2.30 ± 1.10 |
| VV116  (p.o.) | 50 | 0.42 ± 0.14 | 11617 ± 3443 | 24594 ± 1059 | 24739 ± 1028 | 2.15 ± 0.26 | 3.27 ± 1.92 |
| VV116  (p.o.) | 100 | 0.42 ± 0.14 | 24017 ± 6521 | 47799 ± 6545 | 48014 ± 6696 | 2.28 ± 0.53 | 4.25 ± 0.53 |

**Table S7**. **Concentration of X1 in blood of balb/c mice.** Concentration of X1 in blood following oral administration of VV116 at a single dose of 25.0, 50.0 or 100.0 mg/kg in Balb/c mice (N = 3 per group).

|  | VV116, PO, 25.0 mg/kg | | | VV116, PO, 50.0 mg/kg | | | VV116, PO, 100.0 mg/kg | | |
| --- | --- | --- | --- | --- | --- | --- | --- | --- | --- |
| Time | M-1 | M-2 | M-3 | M-4 | M-5 | M-6 | M-7 | M-8 | M-9 |
|  | Conc. (ng/ml) | | | | | | | | |
| 0.25 h | 5800 | 5050 | 4090 | 10150 | 8950 | 13450 | 18000 | 11900 | 31500 |
| 0.5 h | 4290 | 5550 | 4730 | 9100 | 9150 | 15550 | 19550 | 21000 | 25250 |
| 1.0 h | 3715 | 3090 | 3440 | 7550 | 6700 | 6950 | 14850 | 12950 | 13200 |
| 2.0 h | 1930 | 1640 | 2605 | 3925 | 5550 | 4405 | 10950 | 9400 | 6300 |
| 4.0 h | 870 | 930 | 1040 | 1915 | 2360 | 1355 | 4825 | 3255 | 1605 |
| 6.0 h | 197 | 195 | 272 | 397 | 364 | 308 | 1115 | 675 | 313 |
| 8.0 h | 54.0 | 64.0 | 50.5 | 138 | 147 | 135 | 384 | 308 | 228 |
| 24.0 h | 16.1 | BQL | 12.6 | 20.1 | BQL | 11.6 | 55.5 | 33.6 | 10.5 |

BQL = below the quantification limit

**Table S8. Concentration of X1 in liver, lung, and blood in Balb/c mice.** Concentration for X1 in liver, lung, and blood following oral administration of VV116 at a single dose of 100.0 mg/kg in Balb/c mice (N = 6 per group) at 0.25, 2.0, 6.0 and 24 h post-dosing.

| Time point | Tissue | Mice number | | | | | | Average | SD |
| --- | --- | --- | --- | --- | --- | --- | --- | --- | --- |
|  |  | 1 | 2 | 3 | 4 | 5 | 6 |  |  |
| 0.25 h | Liver (ng/g) | 45500 | 29300 | 17000 | 21500 | 32800 | 30200 | 29383 | 9875 |
|  | Lung (ng/g) | 20500 | 11300 | 9620 | 20800 | 18000 | 18700 | 16487 | 4816 |
|  | Blood (ng/ml) | 14000 | 11600 | 9080 | 12900 | 20000 | 17400 | 14163 | 3964 |

| Time point | Tissue | Mice number | | | | | | Average | SD |
| --- | --- | --- | --- | --- | --- | --- | --- | --- | --- |
|  |  | 7 | 8 | 9 | 10 | 11 | 12 |  |  |
| 2.0 h | Liver (ng/g) | 19800 | 19100 | 12600 | 17000 | 12700 | 15700 | 16150 | 3081 |
|  | Lung (ng/g) | 7960 | 8920 | 8470 | 10400 | 6460 | 8840 | 8508 | 1293 |
|  | Blood (ng/ml) | 7820 | 7150 | 6990 | 9430 | 6380 | 6840 | 7435 | 1084 |

| Time point | Tissue | Mice number | | | | | | Average | SD |
| --- | --- | --- | --- | --- | --- | --- | --- | --- | --- |
|  |  | 13 | 14 | 15 | 16 | 17 | 18 |  |  |
| 6.0 h | Liver (ng/g) | 9530 | 13400 | 10600 | 4410 | 8940 | 11500 | 9730 | 3044 |
|  | Lung (ng/g) | 2040 | 2190 | 3010 | 733 | 1400 | 1830 | 1867 | 768 |
|  | Blood (ng/ml) | 1470 | 1780 | 2460 | 917 | 1330 | 1710 | 1611 | 518 |

| Time point | Tissue | Mice number | | | | | | Average | SD |
| --- | --- | --- | --- | --- | --- | --- | --- | --- | --- |
|  |  | 19 | 20 | 21 | 22 | 23 | 24 |  |  |
| 24 h | Liver (ng/g) | 2200 | 1230 | 1170 | 1250 | 1700 | 1400 | 1492 | 396 |
|  | Lung (ng/g) | 131 | 119 | BQL | BQL | 106 | 120 | 119 | 10 |
|  | Blood (ng/ml) | BQL | BQL | BQL | BQL | BQL | BQL | NA | NA |

BQL = below the quantification limit; NA = Not applicable

**Table S9.** **Toxicokinetics parameters for X1 in SD rats.** Calculation of toxicokinetics parameters for X1 after repeated p.o. doses of VV116 in SD rats at Day 1 and Day 14 (N = 4 per group).

| Dose | Gender | Day 1 | | | Day 14 | | |
| --- | --- | --- | --- | --- | --- | --- | --- |
| mg/kg |  | T_max_  (h) | C_max_  (ng/ml) | AUC_0-t_  (ng·h/ml) | T_max_  (h) | C_max_  (ng/ml) | AUC_0-t_  (ng·h/ml) |
| 100 | Male | 0.50 | 5785 | 36057 | 0.88 | 5983 | 38959 |
|  | SD | 0.00 | 962 | 3896 | 0.25 | 1057 | 3309 |
|  | Female | 0.63 | 8755 | 45256 | 0.88 | 10120 | 41357 |
|  | SD | 0.25 | 210 | 4275 | 0.25 | 2187 | 7015 |
|  | Total | 0.56 | 7270 | 40656 | 0.88 | 8051 | 40158 |
|  | SD | 0.18 | 1713 | 6207 | 0.23 | 2724 | 5237 |
| 200 | Male | 0.50 | 12613 | 71568 | 0.63 | 9713 | 67965 |
|  | SD | 0.00 | 2813 | 8288 | 0.25 | 2506 | 6604 |
|  | Female | 0.75 | 15950 | 98734 | 0.67 | 22500 | 96303 |
|  | SD | 0.29 | 2044 | 11194 | 0.29 | 5804 | 11051 |
|  | Total | 0.63 | 14281 | 85151 | 0.64 | 15193 | 80110 |
|  | SD | 0.23 | 2892 | 17146 | 0.24 | 7816 | 17087 |
| 500 | Male | 0.88 | 24600 | 150138 | 0.75 | 36375 | 182397 |
|  | SD | 0.25 | 6918 | 18411 | 0.29 | 9690 | 32539 |
|  | Female | 0.63 | 37050 | 196725 | 1.38 | 43475 | 221683 |
|  | SD | 0.25 | 7688 | 37623 | 1.11 | 18935 | 40223 |
|  | Total | 0.75 | 30825 | 173431 | 1.06 | 39925 | 202040 |
|  | SD | 0.27 | 9494 | 37041 | 0.82 | 14433 | 39851 |
